# Triplane Left Atrial Reservoir Strain in Cardiac Amyloidosis: A Comparative Study with Rhythm-Matched Controls

**DOI:** 10.3390/clinpract16010017

**Published:** 2026-01-16

**Authors:** Marina Leitman, Vladimir Tyomkin, Shmuel Fuchs

**Affiliations:** 1 Department of Cardiology, Shamir Medical Center, Zerifin 70300, Israel; 2 Gray Faculty of Medical and Health Sciences, Tel Aviv University, Tel Aviv 6997801, Israel

**Keywords:** cardiac amyloidosis, transthyretin, left atrial strain, atrial fibrillation, speckle tracking, atrial function, triplane left atrial strain

## Abstract

Background: Cardiac amyloidosis is characterized by progressive myocardial and atrial infiltration, leading to atrial mechanical dysfunction, atrial fibrillation, and thromboembolic complications. Left atrial (LA) strain is an established marker of atrial function; however, data on triplane LA strain in cardiac amyloidosis are limited. Methods: We evaluated transthoracic echocardiographic examinations of 24 patients with cardiac amyloidosis and 24 age-, sex-, rhythm-, and ejection fraction-matched control subjects (9 with atrial fibrillation in each group). Among amyloidosis patients, 21 had transthyretin and 3 had light-chain cardiac amyloidosis. All examinations were performed during 2025. Triplane and biplane LA reservoir strain were assessed using speckle-tracking echocardiography. Two-way analysis of variance tested the effects of disease (amyloidosis vs. control) and rhythm (sinus rhythm vs. atrial fibrillation). Agreement between triplane and biplane measurements was evaluated using Pearson correlation and Bland–Altman analyses. Results: Triplane LA reservoir strain was significantly lower in patients with cardiac amyloidosis compared with controls (6.7 ± 2.7% vs. 16.2 ± 8.3%, *p* < 0.001). Even in sinus rhythm, amyloidosis patients demonstrated markedly impaired LA strain, with mean values similar to those observed in control subjects with atrial fibrillation. Two-way ANOVA revealed significant main effects of disease (F = 68.9, *p* < 0.0001) and rhythm (F = 45.0, *p* < 0.0001), as well as a significant disease–rhythm interaction (F = 26.5, *p* < 0.0001). Triplane and biplane LA strain showed strong correlation (r = 0.90, *p* < 0.0001) with good agreement. Reproducibility was excellent (intra-observer ICC = 0.97; inter-observer ICC = 0.94). Conclusions: Triplane LA reservoir strain is markedly reduced in cardiac amyloidosis and enables comprehensive visualization of atrial mechanical dysfunction. The technique demonstrates high reproducibility and strong agreement with biplane analysis, supporting its use as a complementary tool for characterizing amyloid atriopathy.

## 1. Introduction

The development and increasing availability of disease-modifying therapies for transthyretin cardiac amyloidosis (ATTR-CA) have stimulated intensive research efforts and improved recognition of the disease and its associated clinical risks. Nevertheless, despite the establishment of multimodality diagnostic algorithms aimed at enhancing diagnostic accuracy, cardiac amyloidosis remains frequently underdiagnosed. In a Japanese cohort, the prevalence of cardiac amyloidosis increased markedly with advancing age, reaching 30–40% among patients with left ventricular hypertrophy, low atrial voltage, or both, detected during atrial fibrillation ablation procedures [1].

The diagnostic evaluation of cardiac amyloidosis typically begins with assessment of left ventricular structure and function, as echocardiography remains the first-line imaging modality. However, amyloid atriopathy—reflecting left atrial involvement—is increasingly recognized as a major contributor to disease burden. Amyloid infiltration of the atrial myocardium leads to electrical and mechanical dysfunction, predisposing patients to atrial fibrillation, loss of atrial contractile function, and worsening heart failure due to impaired atrioventricular coupling. The left atrium is affected both by direct amyloid deposition within the atrial wall and by secondary remodeling resulting from advanced left ventricular diastolic dysfunction [2,3]. These processes result in elevated left atrial pressures and severe impairment of atrial mechanical function, a condition commonly referred to as amyloid atriopathy.

Over the past decade, several studies have sought to characterize amyloid atriopathy using speckle-tracking echocardiography of the left atrium, most commonly through monoplane or biplane reservoir strain analysis [4,5,6,7]. More recently, the feasibility of triplane left atrial strain imaging has been demonstrated. Although triplane and biplane strain measurements show strong correlation, triplane analysis allows a more comprehensive visualization of left atrial deformation and regional heterogeneity, providing a more detailed functional “map” of atrial mechanics [8].

Accordingly, in the present study, we evaluated triplane left atrial reservoir strain in patients with cardiac amyloidosis and compared the findings with those of age-, sex-, rhythm-, and ejection fraction–matched control subjects.

## 2. Materials and Methods

### 2.1. Study Population

The study population consisted of 48 subjects, including 24 patients with cardiac amyloidosis and 24 age-, sex-, rhythm-, and ejection fraction–matched control subjects with preserved left ventricular function and no evidence of amyloidosis. The amyloidosis cohort included patients with transthyretin or light-chain cardiac amyloidosis diagnosed according to established clinical, imaging, and laboratory criteria. Control subjects were selected from individuals referred for echocardiographic evaluation who demonstrated normal left ventricular function.

Both amyloidosis patients and control subjects were identified retrospectively. The amyloidosis cohort consisted of consecutive eligible patients, whereas control subjects were selected retrospectively from echocardiography referrals and manually matched for age, sex, cardiac rhythm and left ventricular ejection fraction at the time of examination.

The study population was derived from the same institutional cohort previously evaluated for other echocardiographic parameters; however, the present analysis focused on a distinct endpoint (triplane left atrial strain), and one control subject was replaced to ensure matching by cardiac rhythm. No data or results from the present analysis were included in any previous publication. All echocardiographic examinations included in the study were performed between January and June 2025.

Cardiac amyloidosis was diagnosed according to established international criteria. Transthyretin cardiac amyloidosis was confirmed by the presence of typical echocardiographic features in conjunction with positive bone-avid scintigraphy (Perugini grade ≥2) in the absence of monoclonal protein. Light-chain amyloidosis was diagnosed based on histological confirmation of amyloid deposition with immunohistochemical or immunofluorescent typing. In the present cohort, 21 patients had transthyretin amyloidosis and 3 had light-chain amyloidosis.

### 2.2. Echocardiographic Acquisition and Measurements

All examinations were performed using a Vivid E95 ultrasound system (GE Healthcare, Horten, Norway) equipped with a 1.7–4.0 MHz transducer. Frame rates for speckle-tracking analysis were optimized and maintained at ≥50–90 frames/s whenever feasible. Standard parasternal long- and short-axis views, as well as apical four-, two-, and three-chamber views, were acquired in accordance with current chamber quantification guidelines [9].

Left ventricular diastolic function was assessed according to contemporary recommendations [10], including measurements of mitral E and A velocities, E/A ratio, E-wave deceleration time, septal and lateral tissue Doppler E′ velocities, and the E/E′ ratio.

Left atrial volume was calculated using the biplane area–length method and indexed to body surface area (left atrial volume index, LAVi). Left ventricular mass was calculated using the Devereux formula and indexed to body surface area (left ventricular mass index, LVMi). Relative wall thickness (RWT) was calculated as:

RWT = (2 × PW)/LVEDD

where PW represents posterior wall thickness and LVEDD—left ventricle end-diastolic diameter.

### 2.3. Speckle-Tracking Analysis

All echocardiographic studies were transferred to EchoPAC software (Version 206; GE Healthcare) for offline analysis. Endocardial borders were initially traced using automated speckle-tracking algorithms and manually adjusted when necessary to optimize tracking quality. End-systole was defined by aortic valve closure identified in the apical long-axis view and verified using aortic Doppler recordings.

### 2.4. Global Longitudinal Strain

Peak systolic longitudinal strain was measured for each left ventricular segment, and global longitudinal strain (GLS) was calculated as the average value obtained from the apical four-, two-, and three-chamber views.

### 2.5. Biplane Left Atrial Strain

Biplane left atrial reservoir strain was obtained from the apical four- and two-chamber views using automated software, in accordance with current recommendations [11,12,13].

### 2.6. Triplane Left Atrial Strain

Triplane left atrial strain was assessed using ventricular-dedicated two-dimensional speckle-tracking software (EchoPAC, Version 206; GE Healthcare). Left atrial longitudinal strain was measured separately from apical four-chamber, two-chamber, and three-chamber views by manual tracing of the left atrial endocardial border on high-quality two-dimensional images acquired at frame rates of 50–90 frames/s. Peak left atrial reservoir strain was obtained for each view, and the average of the three values was reported as triplane left atrial reservoir strain. This multi-view two-dimensional approach has been previously described and applied for the assessment of regional left atrial strain heterogeneity [8].

### 2.7. Reproducibility Analysis

To evaluate measurement reproducibility, left atrial reservoir strain was re-measured in 20 randomly selected studies (10 patients with cardiac amyloidosis and 10 control subjects). Inter-observer variability was assessed by two independent observers (M.L. and V.T.) blinded to each other’s results. Intra-observer variability was evaluated by one observer (M.L.) who repeated the measurements at least two weeks later, blinded to the initial results.

Reproducibility was quantified using the two-way random-effects intraclass correlation coefficient for absolute agreement [ICC (2,1)], Bland–Altman analysis (mean bias and 95% limits of agreement), within-subject standard deviation (Sw = SD of differences/√2), and coefficient of variation (CV% = 100 × Sw/mean).

### 2.8. Statistical Analysis

Continuous variables are presented as mean ± standard deviation, and categorical variables as number (percentage). Normality was evaluated using the Shapiro–Wilk test and visual inspection of Q–Q plots. Between-group comparisons were performed using independent-samples t tests or Mann–Whitney U tests, as appropriate. Categorical variables were compared using χ^2^ or Fisher’s exact tests. Associations were evaluated using Pearson or Spearman correlation coefficients. Statistical significance was defined as a two-sided *p* value < 0.05. All analyses were performed using IBM SPSS Statistics version 28.0 (IBM, Armonk, NY, USA).

### 2.9. Ethics

The study was approved by the Helsinki Ethics Committee of Shamir (Assaf Harofeh) Medical Center (Approval No. 137744; 0122-25-ASF(V1), 28 July 2025). Data extraction and analysis began on 1 August 2025, following receipt of this approval. All images were fully de-identified. The study complied with institutional policies and the principles of the Declaration of Helsinki. Given the retrospective and anonymized design, the requirement for written informed consent was waived.

## 3. Results

### 3.1. Baseline Characteristics

Baseline characteristics of the study population are summarized in Table 1.

Patients with cardiac amyloidosis and matched control subjects were comparable in age, sex distribution, body size, and major cardiovascular comorbidities. Atrial fibrillation during echocardiographic acquisition was present in 9 patients (37.5%) in each group, ensuring balanced rhythm distribution. The prevalence of hypertension, diabetes mellitus, chronic kidney disease, ischemic heart disease, chronic pulmonary disease, and prior atrial fibrillation did not differ significantly between groups. Functional status, as reflected by New York Heart Association (NYHA) class, was modestly worse in the amyloidosis group (2.8 ± 0.5 vs. 2.3 ± 0.9; *p* < 0.04). Among patients with cardiac amyloidosis, 21 had transthyretin amyloidosis and 3 had light-chain amyloidosis.

#### Echocardiographic Characteristics

Echocardiographic findings are presented in Table 2.

Left ventricular wall thicknesses and mass index were significantly higher in patients with cardiac amyloidosis compared with controls, consistent with myocardial infiltration. Although left ventricular ejection fraction was preserved in both groups, global longitudinal strain was markedly reduced in amyloidosis (−10.2 ± 2.6% vs. −20.1 ± 2.4%, *p* < 0.0001), indicating severe impairment of longitudinal myocardial mechanics. Left ventricular end-diastolic diameter was modestly smaller in amyloidosis, while diastolic filling indices demonstrated higher E/E′ ratios, consistent with elevated filling pressures.

Left atrial reservoir strain was markedly impaired in patients with amyloidosis using both biplane and triplane assessments (6.4 ± 2.5% and 6.7 ± 2.7%, respectively), compared with control subjects (15–16%; *p* = 0.0001 for both). Tricuspid annular peak systolic velocity was also significantly lower in amyloidosis, reflecting impaired right ventricular longitudinal function, whereas estimated pulmonary artery pressures and left atrial volume index were similar between groups. Age was not significantly correlated with triplane left atrial reservoir strain in the overall cohort, indicating that observed differences in atrial strain were not driven by age.

### 3.2. Effect of Disease Status and Cardiac Rhythm on Triplane LA Strain

**Figure 1:**
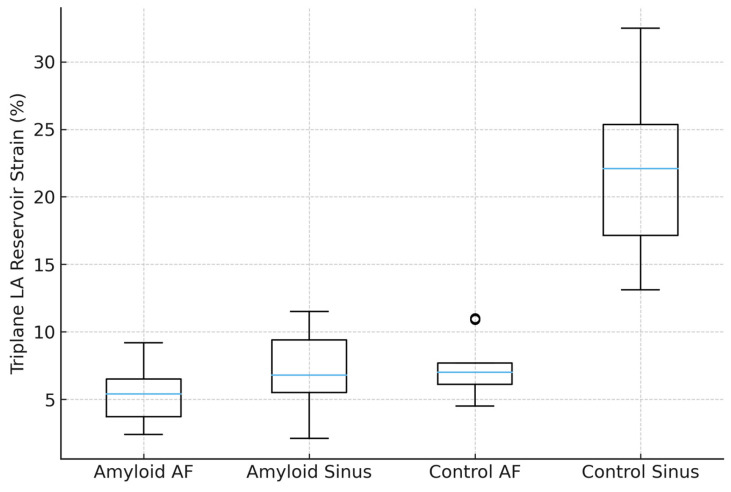
Distribution of triplane left atrial (LA) reservoir strain among study groups. Boxplot illustrating triplane LA reservoir strain in patients with cardiac amyloidosis and in controls, stratified by cardiac rhythm during echocardiographic examination (sinus rhythm vs. atrial fibrillation). LA strain was lowest in amyloidosis patients with atrial fibrillation and highest in controls with sinus rhythm, demonstrating a graded reduction across the four groups (Amyloidosis AF < Amyloidosis Sinus < Control AF < Control Sinus). Boxes indicate interquartile range, horizontal lines the median, and whiskers the 5–95th percentiles, and circles denote outliers.

Triplane left atrial reservoir strain demonstrated a graded distribution across the four disease–rhythm subgroups (Table 3, Figure 1).

The lowest strain values were observed in patients with cardiac amyloidosis and atrial fibrillation (5.5 ± 2.2%), followed by amyloidosis patients in sinus rhythm (7.4 ± 2.7%) and control subjects with atrial fibrillation (7.4 ± 2.1%). Control subjects in sinus rhythm exhibited preserved LA strain (21.5 ± 5.8%).

Two-way analysis of variance confirmed significant main effects of disease (F = 68.9, *p* < 0.0001) and rhythm (F = 45.0, *p* < 0.0001), as well as a significant disease-by-rhythm interaction (F = 26.5, *p* < 0.0001; Table 4), indicating that the impact of atrial fibrillation on LA strain was more pronounced in patients with cardiac amyloidosis.

### 3.3. Post Hoc Group Comparisons

Post hoc pairwise comparisons (Table 5) demonstrated a progressive decline in triplane LA reservoir strain from control subjects in sinus rhythm to patients with cardiac amyloidosis and atrial fibrillation.

All pairwise comparisons between amyloidosis and control groups, as well as between sinus rhythm and atrial fibrillation within each disease category, remained significant after Holm correction, with the exception of the comparison between amyloidosis patients in sinus rhythm and control subjects with atrial fibrillation (adjusted *p* = 0.12).

### 3.4. Representative Triplane LA Strain Patterns

**Figure 2:**
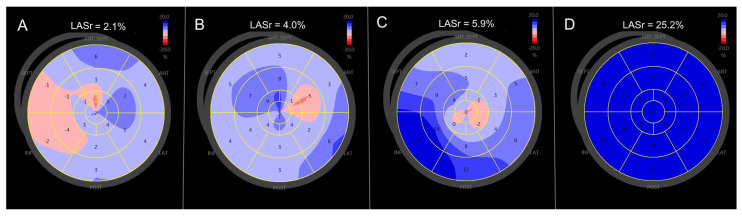
Triplane left atrial reservoir strain in representative study patients. (**A**) Patient with cardiac amyloidosis and atrial fibrillation demonstrating markedly reduced and heterogeneous strain values. (**B**) Patient with cardiac amyloidosis in sinus rhythm showing slightly higher—but still substantially impaired and heterogeneous—LA strain compared with panel (**A**). (**C**) Control patient with atrial fibrillation demonstrating reduced and heterogeneous LA strain, with a visual appearance similar to panel (**B**). (**D**) Control patient in sinus rhythm exhibiting normal, uniform LA reservoir strain. Color-coded strain maps illustrate qualitative differences in left atrial deformation across rhythm and disease categories, consistent with the quantitative group comparisons. Lower strain values are represented by red hues (approximately −20%), whereas higher strain values are depicted by intense blue hues (approximately +20%).

Representative examples of triplane left atrial reservoir strain maps are shown in Figure 2.

Patients with cardiac amyloidosis and atrial fibrillation exhibited markedly reduced and heterogeneous LA strain, whereas amyloidosis patients in sinus rhythm showed modestly higher but still substantially impaired and heterogeneous deformation. Control subjects with atrial fibrillation demonstrated reduced strain with a visual appearance similar to that observed in amyloidosis patients in sinus rhythm. In contrast, control subjects in sinus rhythm displayed normal, uniform LA reservoir strain. These qualitative examples correspond closely with the quantitative group-level findings, particularly the similar mean strain values observed in amyloidosis patients in sinus rhythm and control subjects with atrial fibrillation.

### 3.5. Agreement Between Triplane and Biplane LA Strain

**Figure 3:**
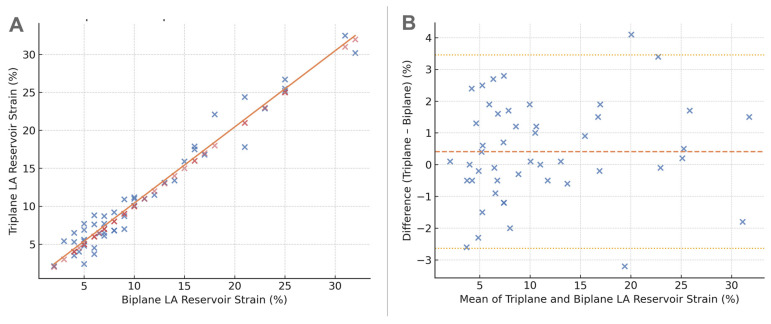
Agreement between triplane and biplane LA reservoir strain. (**A**) Scatterplot showing the relationship between triplane LA reservoir strain and biplane LA reservoir strain in all participants. The orange regression line illustrates the close correlation between the two methods (r = 0.94, *p* < 0.0001). (**B**) Bland–Altman plot showing the difference between triplane and biplane LA reservoir strain. The mean bias (red dashed line) represents the average difference (Triplane − Biplane), and the dotted orange lines denote the 95% limits of agreement. Most differences cluster near zero, indicating good agreement and minimal systematic bias.

Triplane and biplane LA reservoir strain measurements showed a strong linear relationship (Pearson r = 0.94, *p* < 0.0001; 95% CI, 0.88–0.97). The correlation scatterplot (Figure 3A) demonstrated a near one-to-one relationship with minimal dispersion around the regression line. Bland–Altman analysis (Figure 3B) revealed a small mean bias of −0.5%, with 95% limits of agreement from −4.2% to +3.2%, indicating minimal systematic differences and no proportional bias across the measurement range.

### 3.6. Correlations with Ventricular Function and Filling Parameters

**Figure 4:**
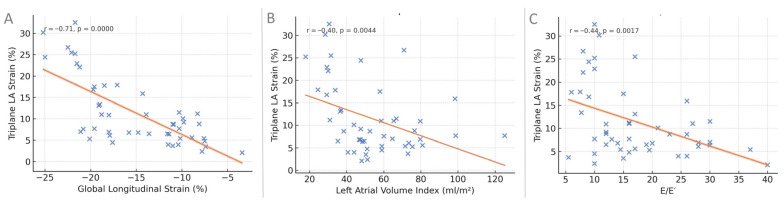
Correlation of triplane LA reservoir strain with selected echocardiographic parameters. (**A**) Relationship between triplane LA reservoir strain and left ventricular global longitudinal strain. (**B**) Relationship between triplane LA reservoir strain and left atrial volume index. (**C**) Relationship between triplane LA reservoir strain and the E/E′ ratio. In each panel, blue dots represent individual patients and the orange line indicates the linear regression fit.

Triplane LA reservoir strain demonstrated a strong inverse correlation with left ventricular global longitudinal strain (r = −0.71, *p* < 0.0001), indicating that worsening ventricular deformation was associated with impaired atrial reservoir function (Table 6, Figure 4). Moderate negative correlations were also observed with left atrial volume index (r = −0.40, *p* = 0.004) and E/E′ ratio (r = −0.44, *p* = 0.002), suggesting that reduced atrial strain accompanies both atrial dilatation and elevated filling pressures.

### 3.7. Reproducibility Analysis

**Figure 5:**
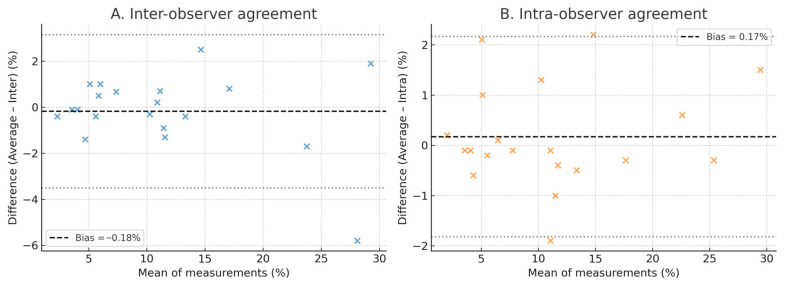
Bland–Altman plots for reproducibility of three-plane left atrial reservoir strain. (**A**) Inter-observer agreement between two independent observers. (**B**) Intra-observer agreement between two repeated measurements by the same observer. The central dashed line represents the mean bias, and the dotted lines indicate the 95% limits of agreement (mean bias ± 1.96 × standard deviation of the differences). Each point represents one subject’s triplane left atrial reservoir strain value. Values are expressed in percent strain (%). Both inter- and intra-observer comparisons demonstrated excellent reproducibility, with negligible systematic bias and narrow limits of agreement across the full range of strain values.

Inter-observer and intra-observer reproducibility results are presented in Table 7 and Figure 5. Inter-observer reproducibility was excellent (ICC = 0.98, CV ≈ 10.6%), and intra-observer reproducibility was near-perfect (ICC = 0.99, CV ≈ 6.5%). Similar reproducibility metrics were observed when amyloidosis and control subgroups were analyzed separately. Bland–Altman analyses demonstrated negligible systematic bias and narrow limits of agreement across the full range of strain values.

## 4. Discussion

In this study, we evaluated triplane left atrial reservoir strain in patients with cardiac amyloidosis compared with age-, sex-, and rhythm-matched control subjects. The principal finding is that triplane LA reservoir strain is markedly reduced in amyloidosis patients even when they are in sinus rhythm, with strain values similar to those observed in control subjects with atrial fibrillation. These findings indicate the presence of a severe atrial cardiomyopathy in cardiac amyloidosis that is largely independent of rhythm status.

### 4.1. Relation to Previous Studies

Our findings build upon and extend prior investigations using monoplane or biplane LA strain assessment in cardiac amyloidosis. Previous studies have consistently demonstrated that LA reservoir, conduit, and contractile strain components are significantly impaired in amyloidosis and correlate with left ventricular dysfunction, disease severity, prognosis, and thromboembolic risk [4,7,14].

In particular, Inoue et al. [15] showed that among patients with left ventricular hypertrophy, LA reservoir strain provided diagnostic utility in differentiating cardiac amyloidosis from other causes of hypertrophy (area under the curve ≈0.81). Reduced LA strain has also been reported in cardiac amyloidosis compared with Fabry disease [16] and hypertensive heart disease [17].

While the concept of amyloid-related LA myopathy is therefore well established, the present study adds incremental value by applying a triplane imaging approach. Triplane LA strain enables not only quantitative assessment of atrial reservoir function but also visualization of regional heterogeneity in atrial deformation and mechanics. As illustrated in Figure 2, this qualitative visualization provides insight into the pathophysiologic mechanisms linking amyloid infiltration, atrial dysfunction, atrial fibrillation, and thromboembolic vulnerability.

### 4.2. Pathophysiologic Implications

The marked reduction in triplane LA reservoir strain observed in amyloidosis patients—even in sinus rhythm—supports the concept that the left atrium is affected early and profoundly in the disease course. This impairment likely reflects a combination of direct amyloid infiltration of the atrial wall, elevated left atrial pressure secondary to restrictive ventricular physiology, and loss of atrial compliance and contractile reserve. These observations are consistent with histopathologic evidence of atrial amyloid deposition and imaging studies demonstrating increased atrial stiffness and mechanical dispersion in ATTR-CA [2,3].

Notably, amyloidosis patients in sinus rhythm exhibited triplane LA strain values similar to those of control subjects with atrial fibrillation, underscoring the severity of atrial mechanical dysfunction in amyloid cardiomyopathy. This finding may help explain the high prevalence of atrial fibrillation, atrial enlargement, thromboembolic events, and progressive heart failure in this population [18,19,20]. Although some post hoc pairwise comparisons did not reach statistical significance, the strong disease–rhythm interaction demonstrated by two-way ANOVA highlights the disproportionate adverse impact of atrial fibrillation on LA mechanics in amyloidosis compared with controls.

The regional heterogeneity observed on triplane LA strain maps may reflect patchy amyloid infiltration, fibrotic remodeling, and intra-atrial mechanical dispersion—features that constitute established substrates for atrial arrhythmias and intracavitary thrombus formation (Figure 2).

### 4.3. Clinical and Diagnostic Implications

From a clinical perspective, our findings suggest that triplane LA strain may serve as an additional imaging biomarker for assessing the burden of atrial involvement in cardiac amyloidosis. While echocardiographic evaluation has traditionally focused on left ventricular wall thickness and strain, atrial strain provides complementary information regarding atrial remodeling, mechanical dysfunction, and potential arrhythmic and thromboembolic risk.

Incorporating triplane LA strain into the diagnostic work-up of cardiac amyloidosis may improve sensitivity for detecting early atrial involvement, particularly in patients who remain in sinus rhythm and do not yet exhibit overt atrial enlargement. In the present cohort, triplane LA reservoir strain values below approximately 8% were predominantly observed in amyloidosis patients, whereas values exceeding 15–20% were typical of control subjects in sinus rhythm. These thresholds should be considered exploratory and require validation in larger prospective studies.

Although biplane LA strain reduction in amyloidosis has been consistently reported, the principal contribution of the present study lies in demonstrating the utility of triplane LA strain for visualizing regional heterogeneity of atrial mechanics. By integrating deformation data from three orthogonal apical views, the triplane approach provides an intuitive representation of atrial mechanical dispersion and patchy dysfunction that may be less apparent with conventional mono- or biplane techniques.

### 4.4. Reproducibility and Feasibility

An important strength of this study is the excellent reproducibility of triplane LA strain measurements. Both inter- and intra-observer analyses demonstrated high intraclass correlation coefficients, low coefficients of variation, and narrow Bland–Altman limits of agreement, indicating robust measurement reliability. Importantly, reproducibility was maintained in both sinus rhythm and atrial fibrillation, supporting the feasibility of triplane LA strain assessment across different rhythm states. These findings are consistent with prior reproducibility studies of LA strain [12,21] and support the potential applicability of triplane LA strain in clinical practice, longitudinal follow-up, and multicenter research settings.

### 4.5. Strengths and Limitations

The strengths of our study include the use of a carefully matched control group (age, sex, rhythm, and ejection fraction) and the application of a triplane imaging approach that enables comprehensive atrial assessment with both quantitative and qualitative components.

Several limitations should be acknowledged. First, this was a single-center, cross-sectional observational study, precluding causal inference and outcome assessment. Second, although rhythm matching was performed, subgroup sizes were modest. Third, clinical outcomes such as incident atrial fibrillation, thromboembolic events, and mortality were not evaluated. Given the small number of patients with light-chain amyloidosis, meaningful subgroup analyses by amyloid subtype were not feasible. Finally, evaluation of the independent effects of medical therapy or biomarker levels on triplane LA strain was beyond the scope of this study.

### 4.6. Future Directions

Future studies should investigate the prognostic value of triplane LA strain in larger prospective cohorts, including endpoints such as atrial fibrillation onset, thromboembolic events, heart failure hospitalization, and mortality. Comparative analyses of mono-, bi-, and triplane LA strain may further clarify the incremental value of each technique. Integration of atrial functional imaging with structural modalities such as cardiac MRI or PET may provide additional mechanistic insight. Longitudinal assessment of triplane LA strain during disease-modifying therapy (e.g., tafamidis) may also help determine whether atrial mechanical dysfunction is reversible and clinically meaningful [22,23,24,25].

## 5. Conclusions

In conclusion, triplane LA reservoir strain is profoundly reduced in patients with cardiac amyloidosis even in sinus rhythm, reaching values comparable to those observed in control subjects with atrial fibrillation—findings consistent with severe atrial cardiomyopathy. The ability of triplane imaging to visualize regional atrial mechanical heterogeneity offers additional pathophysiologic insight and may enhance risk stratification in cardiac amyloidosis. Further studies are warranted to define its prognostic and therapeutic implications.

## Figures and Tables

**Table 1:** Baseline Characteristics of the Study Population.

Variable	Amyloidosis (*n* = 24)	Controls (*n* = 24)	*p*-Value
Patients	24	24	1.0
Male sex, *n* (%)	20 (83.3)	20 (83.3)	1.0
Age, years	84.8 ± 5	81.9 ± 7.9	0.1
Atrial fibrillation during echocardiography, *n* (%)	9.0 (37.5)	9.0 (37.5)	1.0
Weight, kg	71.1 ± 11.1	77.7 ± 14.2	0.1
Height, cm	166.3 ± 9.2	169.7 ± 7.6	0.2
BMI, kg/m^2^	25.5 ± 3	26.9 ± 4.4	0.3
BSA, m^2^	1.8 ± 0.2	1.8 ± 0.2	0.1
Hypertension, *n* (%)	17(70.8)	20(83.3)	0.4
Diabetes mellitus, *n* (%)	9 (37.5)	9 (37.5)	1.0
Chronic kidney disease, *n* (%)	15 (62.5)	10 (41.7)	0.9
Ischemic heart disease, *n* (%)	6 (25.0)	6 (25.0)	1.0
Chronic pulmonary disease, *n* (%)	6 (25.0)	10 (41.7)	0.5
History of atrial fibrillation, *n* (%)	16 (66.7)	13 (54.2)	0.8
Malignancy, *n* (%)	5 (20.8)	9 (37.5)	0.4
NYHA, functional class	2.8 ± 0.5	2.3 ± 0.9	<0.04

Values are expressed as mean ± SD or *n* (%). Amyloidosis = cardiac amyloidosis group; NYHA = New York Heart Association functional class.

**Table 2:** Echocardiographic Characteristics of Patients with Cardiac Amyloidosis (Group 1) and Controls (Group 2).

Variable	Amyloidosis (*n* = 24)	Controls (*n* = 24)	*p*-Value
Heart rate, beats/min	72 ± 12.6	73.6 ± 12.1	0.67
Ejection fraction, %	53.8 ± 7.3	55.8 ± 4.2	0.26
Global longitudinal strain, %	−10.2 ± 2.6	−20.1 ± 2.4	<0.0001
Left ventricular mass index, g/m^2^	145.0 ± 30.1	117.2 ± 62.1	0.06
Regional wall thickness	0.66 ± 0.13	0.47 ± 0.06	<0.0001
Interventricular thickness, cm	1.7 ± 0.3	1.4 ± 0.3	<0.0001
Posterior wall thickness, cm	1.3 ± 0.2	1.1 ± 0.2	<0.0001
Left ventricle end diastolic diameter, cm	4.2 ± 0.5	4.6 ± 0.6	<0.03
Left ventricle end systolic diameter, cm	2.7 ± 0.5	2.8 ± 0.6	0.60
E/E′	20.1 ± 8.4	14.1 ± 7.1	0.01
E deceleration, msec	189.2 ± 53.5	194.0 ± 65.4	0.80
Left atrial volume index, mL/m^2^	54.9 ± 15.8	53.3 ± 26.1	0.80
Biplane left atrial strain, %	6.4 ± 2.5	15.7 ± 8.1	0.0001
Triplane left atrial strain, %	6.7 ± 2.7	16.2 ± 8.3	0.0001
Tricuspid annulus peak systolic velocity, cm/s	1.5 ± 0.4	2.1 ± 0.4	0.0001
Pulmonary artery pressure, mm Hg	44.6 ± 15.3	41.7 ± 11.5	0.50

Values are mean ± SD. Abbreviations: E/E′ = ratio of transmitral early diastolic velocity to annular early diastolic velocity.

**Table 3:** Triplane left atrial reservoir strain by disease status and rhythm.

Group	Number	Triplane LA Reservoir Strain (%) Mean ± SD
Amyloidosis + atrial fibrillation	9	5.5 ± 2.2
Amyloidosis + sinus rhythm	15	7.4 ± 2.7
Controls + atrial fibrillation	9	7.4 ± 2.1
Controls + sinus rhythm	15	21.5 ± 5.8

**Table 4:** Two-way analysis of variance (ANOVA) of triplane left atrial (LA) reservoir strain according to disease status (cardiac amyloidosis vs. controls) and cardiac rhythm (atrial fibrillation vs. sinus rhythm).

Effect	Sum Sq	df	F	*p*-Value
Disease	1084.0	1	68.9	<0.0001
Rhythm	707.9	1	45.0	<0.0001
Disease × Rhythm	416.3	1	26.5	<0.0001
Residual	692.3	44		

Abbreviations: LA = left atrial; ANOVA = analysis of variance; df = degrees of freedom; F = Fisher’s F-statistic; *p* = probability value; Disease = main effect of cardiac amyloidosis vs. control; Rhythm = main effect of atrial fibrillation vs. sinus rhythm; Disease × Rhythm = interaction term between disease and rhythm; Residual = within-group (error) variance.

**Table 5:** Post hoc pairwise comparisons of triplane left atrial (LA) reservoir strain among study groups.

Comparison	t (Welch)	*p* (Raw)	*p* (Holm-Adjusted)	Significant
Amyloidosis AF vs. Control Sinus	−11.2	<0.0001	<0.0001	Yes
Amyloidosis AF vs. Control AF	−5.9	<0.0001	0.0002	Yes
Amyloidosis AF vs. Amyloidosis Sinus	−4.8	0.00003	0.0004	Yes
Amyloidosis Sinus vs. Control Sinus	−6.5	<0.0001	0.0001	Yes
Control AF vs. Control Sinus	−3.8	0.0004	0.003	Yes
Amyloidosis Sinus vs. Control AF	−2.2	0.04	0.12	No

LA = left atrial; AF = atrial fibrillation; t = Welch’s t-statistic; *p* = probability value; Holm = Holm adjustment for multiple comparisons.

**Table 6:** Correlation between triplane left atrial reservoir strain and selected echocardiographic parameters.

Variable	N	r	*p*	95% CI Low	95% CI High
GLS	48	−0.71	<0.0001	−0.83	−0.54
LAVi	48	−0.40	0.004	−0.62	−0.14
E/E′	48	−0.44	0.002	−0.64	−0.18

Abbreviations: GLS = global longitudinal strain; LAVi = left atrial volume index; E/E′ = ratio of mitral inflow velocity to annular early diastolic velocity; CI = confidence interval.

**Table 7:** Inter- and Intra-Observer Variability of 3-Plane Left Atrial Reservoir Strain.

Subgroup	Type	*n*	ICC (2,1)	Bias (A–B)	95% Limits of Agreement	Within-Subject SD	CV (%)	Mean (%)
All patients, *n* = 20	Inter-observer	20	0.98	−0.18	−3.50 to +3.15	1.20	10.6	11.3
All patients, *n* = 20	Intra-observer	20	0.99	0.17	−1.82 to +2.16	0.72	6.5	11.1
Amyloidosis, *n* = 10	Inter-observer	10	0.98	0.02	−1.43 to +1.47	0.52	8.2	6.4
Amyloidosis, *n* = 10	Intra-observer	10	0.98	−0.21	−1.64 to +1.22	0.52	8.0	6.5
Controls, *n* = 10	Inter-observer	10	0.96	−0.37	−4.95 to +4.20	1.65	10.2	16.2
Controls, *n* = 10	Intra-observer	10	0.99	0.55	−1.71 to +2.81	0.82	5.2	15.8

Abbreviations: ICC = intraclass correlation coefficient; SD = standard deviation; CV = coefficient of variation.

## Data Availability

The raw data supporting the conclusions of this article will be made available by the authors on request.
